# Multigenerational epigenetic effects of nicotine on lung function

**DOI:** 10.1186/1741-7015-11-27

**Published:** 2013-02-04

**Authors:** Frances M Leslie

**Affiliations:** 1 Department of Pharmacology, School of Medicine, University of California, Irvine, CA 92697, USA

**Keywords:** development, DNA methylation, histone acetylation, nicotine replacement therapy (NRT), peroxisome proliferator-activated receptor-γ (PPARγ), smoking, tobacco

## Abstract

A recent preclinical study has shown that not only maternal smoking but also grandmaternal smoking is associated with elevated pediatric asthma risk. Using a well-established rat model of *in utero *nicotine exposure, Rehan *et al*. have now demonstrated multigenerational effects of nicotine that could explain this 'grandmother effect'. F1 offspring of nicotine-treated pregnant rats exhibited asthma-like changes to lung function and associated epigenetic changes to DNA and histones in both lungs and gonads. These alterations were blocked by co-administration of the peroxisome proliferator-activated receptor-γ agonist, rosiglitazone, implicating downregulation of this receptor in the nicotine effects. F2 offspring of F1 mated animals exhibited similar changes in lung function to that of their parents, even though they had never been exposed to nicotine. Thus epigenetic mechanisms appear to underlie the multigenerational transmission of a nicotine-induced asthma-like phenotype. These findings emphasize the need for more effective smoking cessation strategies during pregnancy, and cast further doubt on the safety of using nicotine replacement therapy to reduce tobacco use in pregnant women.

Please see related article: http://www.biomedcentral.com/1741-7015/10/129

## Background

**Figure 1:**
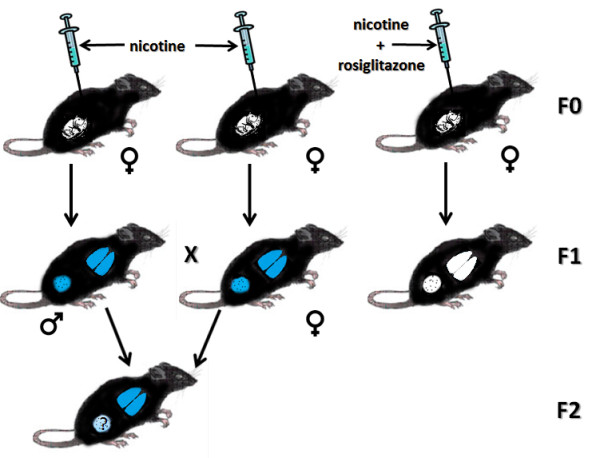
**Multigenerational transmission of nicotine-induced effects**. The diagram illustrates the experimental design and findings of Rehan *et al. *[4]. Pregnant dams (F0 generation) are injected with nicotine or nicotine + rosiglitazone. The lungs and gonads of both male and female offspring (F1 generation) of nicotine-treated dams exhibit epigenetic changes, and the lungs show an asthma-like functional phenotype (blue nicotine-induced changes). These nicotine effects are not seen in the offspring of animals treated with nicotine + rosiglitazone. Offspring of F1 mated pairs (F2 generation) exhibit the same nicotine-induced changes to lung function as their parents, even though they were not exposed to drug. It is not yet known whether the F2 offspring continue to exhibit alterations to the germ cell epigenome.

The negative health effects of tobacco use in adult smokers are well established [1]. On average, smoking leads to more than 400,000 premature deaths in the United States each year, with an overall decrease in life expectancy of 14 years. The major adverse health consequences of smoking include cancer, cardiovascular disease and respiratory disorders. Since many women continue to smoke during pregnancy, the negative impact of tobacco can begin before birth [2]. Maternal smoking is now the single most important preventable risk factor for Sudden Infant Death Syndrome, which results from developmental delays in the neural control of cardiopulmonary function [1,2]. Children of smokers are also more prone to respiratory diseases, such as asthma. One surprising finding is that a grandmother's tobacco use is associated with increased risk of early childhood asthma, even if the mother did not smoke while pregnant [3]. Rehan *et al. *[4] have recently used a well-established rat model of *in utero *nicotine exposure to determine the possible mechanisms underlying this clinical observation (Figure 1). They found that maternal nicotine exposure exerted adverse effects on lung development, not only for the immediate offspring but also for the next generation. They also identified epigenetic mechanisms involved in this multigenerational transmission. This paper will review these groundbreaking findings and discuss their potential clinical implications.

## Transgenerational transmission of nicotine effects

Within recent years, fundamental assumptions about genetic inheritance have been revisited [5,6]. In addition to classical Mendelian genetics, the environment has been shown to contribute to inherited characteristics by placing epigenetic tags on DNA or associated histones that result in modified gene expression. In particular, the prenatal environment can result in reprogramming of the epigenome, as demonstrated by Rehan *et al. *[4]. They showed that acute daily injections of nicotine throughout pregnancy led to epigenetic modifications of lung tissue in the F1 offspring (Figure 1), with a resulting asthma-like phenotype. When F1 rats were mated, similar changes in lung function were observed in their F2 offspring, even though they had never been exposed to nicotine. Nicotine induction of the asthma phenotype was found to result from a downregulation of mesenchymal peroxisome proliferator-activated receptor-γ (PPARγ), which plays a critical role in the development, homeostasis and repair of the lung [7]. Rosiglitazone, a PPARγ agonist, completely prevented the alterations in lung function, and in H3 acetylation of lung histones, when co-administered with nicotine to the pregnant dam [4].

Although the F2 rats had never been exposed to nicotine, their primordial germ cells were potential targets while the F1 parents were *in utero*. The finding of nicotine-induced epigenetic changes in both ovarian and testicular tissues from F1 generation rats provides support for this as a possible mechanism for functional changes observed in F2 offspring. Whereas H4 histone acetylation was increased in the gonads of both sexes, DNA methylation was increased in testes but decreased in ovaries. All of these epigenetic changes were eliminated by rosiglitazone, implicating the downregulation of PPARγ as a more universal mechanism of nicotine-induced changes to the epigenome, impacting germ cells as well as lung tissue.

*In utero *nicotine exposure resulted in alterations to both the somatic and germ cell epigenome. However, the nicotine-induced germ cell epigenetic changes were only examined in the gonads of F1 offspring. The true test of whether nicotine can induce permanent epigenetic changes to the germline, with resulting transgenerational genetic inheritance, will require studies that look at germ cells in the F2 offspring and lung function in subsequent F3 and F4 generations. This issue notwithstanding, however, this preclinical study is critically important in that it provides the first experimental evidence for multigenerational effects of *in utero *nicotine exposure. Furthermore, it provides a conceptual framework with which to understand the novel clinical observation that grandmothers' smoking patterns are as important as that of the mother in determining pediatric lung function [3].

## Future directions

Given the significant health risks to the offspring, effective smoking cessation strategies during pregnancy are clearly needed. Whereas behavioral therapy does not involve drug exposure to the fetus, it is not always effective. This has led some practitioners to advocate the use of nicotine replacement therapy (NRT) as a smoking cessation aid for pregnant women since it may reduce the risk of low birth weight and preterm delivery [8]. However, the clinical community is divided on this recommendation because of continued concerns about efficacy and safety [9,10]. Preclinical investigators have long argued that nicotine is a developmental teratogen and should not be used as a treatment for pregnant smokers [2,11]. The current finding of multigenerational effects of *in utero *nicotine exposure will provide critical support for this view.

Another issue that requires further assessment is whether nicotine can induce more generalized epigenetic changes, not only as a result of *in utero *exposure. Primordial germ cells are also exposed to nicotine in non-pregnant female and male smokers, and may undergo epigenetic changes that are transmitted to future progeny. Support for this concept comes from a recent study that found cigarette smoke to induce specific differences in the spermatozoal microRNA content of human smokers as compared to non-smokers [12]. The microRNAs that were differentially impacted mediate pathways required for healthy sperm and normal embryo development, suggesting a possible adverse impact of smoking on these processes. Smoking may also produce somatic changes in the developing brain during its critical adolescent period. Nearly all tobacco use begins in childhood and adolescence, with almost 90% of adult smokers reporting that they started smoking by the age of 18 [13]. A growing clinical and preclinical literature suggests that adolescent nicotine exposure produces unique and long-term changes in neural structure, function and resulting behavior [11,14]. Similar PPARγ-mediated epigenetic mechanisms to those identified by Rehan and colleagues [4] may underlie these unique developmental effects of nicotine during adolescence.

Findings of widespread nicotine-induced changes to the epigenome should stimulate further, more aggressive, efforts to restrict youth access to tobacco products. They may also lead to possible therapeutic strategies to prevent or reverse the adverse impact of nicotine exposure on the developing brain and periphery.

## Abbreviations

NRT: nicotine replacement therapy; PPARγ: peroxisome proliferator-activated receptor-γ

## Competing interests

The author declares that they have no competing interests.

## Author's information

FML is Professor of Pharmacology and Dean of the Graduate Division at the University of California, Irvine. She has published extensively on the effects of nicotine on the developing brain.

## Pre-publication history

The pre-publication history for this paper can be accessed here:

http://www.biomedcentral.com/1741-7015/11/27/prepub
